# Rotating Gate-Driven Solution-Processed Triboelectric Transistors

**DOI:** 10.3390/s22093309

**Published:** 2022-04-26

**Authors:** Hyunji Shin, Dae Yu Kim

**Affiliations:** 1 Department of Electrical and Computer Engineering, Inha University, Incheon 22212, Korea; hshin@inha.ac.kr; 2 Center for Sensor Systems, College of Engineering, Inha University, Incheon 22212, Korea

**Keywords:** triboelectric effect, transistor, energy harvesting, poly(tetrafluoroethylene) (PTFE), rotating gate transistor (RGT), triboelectric nanogenerator (TENG)

## Abstract

Among various energy harvesting technologies, triboelectricity is an epoch-making discovery that can convert energy loss caused by the mechanical vibration or friction of parts into energy gain. As human convenience has emerged as an important future value, wireless devices have attracted widespread attention; thus, it is essential to extend the duration and lifespan of batteries through energy harvesting or the application of self-powered equipment. Here, we report a transistor, in which the gate rotates and rubs against the dielectric and utilizes the triboelectricity generated rather than the switching voltage of the transistor. The device is a triboelectric transistor with a simple structure and is manufactured using a simple process. Compared to that at the stationary state, the output current of the triboelectric transistor increased by 207.66 times at the maximum rotation velocity. The approach reported in this paper could be an innovative method to enable a transistor to harness its own power while converting energy loss in any rotating object into harvested energy.

## 1. Introduction

As human convenience has emerged as one of the most important future values, in recent decades, modern technology has focused on the development of numerous wireless Internet of Things (IoT) systems [1,2,3]. However, such wireless devices depend on the duration and lifespan of batteries to continuously provide power to the device; thus, they require recharging and replacement [4,5]. For example, electric vehicles, which are expected to dominate the green automobile industry in the future, currently require more than 100 sensors, and the energy consumption of these future vehicles is expected to increase as the technology advances [6,7]. Accordingly, energy-harvesting devices, such as piezoelectric nanogenerators, triboelectric nanogenerators (TENGs), and solar cells, are attracting attention and have emerged as a very promising technology for collecting surrounding energy that is wasted in our living environment, various devices, or electric vehicles [8,9,10,11,12]. Particularly, triboelectricity, which has been investigated for several years and was previously considered a harmful phenomenon, has attracted significant research attention in various fields such as energy harvesting as well as health monitoring since the invention of TENG by Fan et al. [13] in 2012 owing to its small-scale, easy fabrication, and high efficiency [14,15,16,17,18].

Poly(tetrafluoroethylene) (PTFE) is primarily considered a negative triboelectric material in TENGs owing to its extremely low triboelectric coefficient and has attracted widespread attention in various applications, including insulation materials, automotive parts, and semiconductor manufacturing facilities because of its attractive combination of chemical resistance and excellent noncohesive properties [19,20,21]. However, the low-dielectric constant (low-k; 2.1) of PTFE has limited its application in general transistors, including field-effect transistors (FETs); moreover, there are significantly fewer studies on triboelectric-based transistors compared to FETs [22,23,24]. In addition, research on triboelectric-based transistors mainly adopts a structure wherein TENG is grafted to or the triboelectric material makes contact with the gate electrode of the transistor [25,26,27,28]. Although this has emerged as a groundbreaking method for harnessing the energy harvested by triboelectricity, it is a process-intensive method, as it merely involves the addition of TENG to an existing transistor. This study proposes a triboelectric transistor with a very simple structure, in which the gate rubs itself against the dielectric.

The developed rotating gate transistor (RGT) is a transistor that utilizes the triboelectricity generated by the friction between a rotating gate and a dielectric layer. In this study, poly(3-hexylthiophene-2,5-diyl) (P3HT), which is widely used in organic semiconductor-based FETs or organic solar cells, was utilized as a semiconductor layer on the opposite side of the friction surface. Molecules of P3HT interact by van der Waals forces, which are significantly weaker than covalent bonds, thus enabling excellent solubility at low temperatures and flexible device implementations [29,30,31]. Consequently, the proposed device was capable of direct replacement from FET to RGT, and various types of semiconductors can be applied to the active layer of our device. The proposed transistor is a fairly innovative device that can be directly applied as a gate-free structure to a moving object where friction is possible. Moreover, our performance evaluation results may help pioneer a new field of convergence of RGT and FET in the future.

## 2. Experimental Details

**Figure 1:**
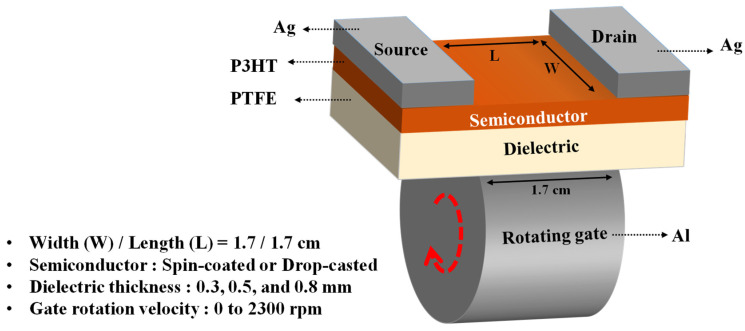
Schematic illustration of the triboelectric transistor with a rotating gate. The gate rotates in the direction of the red dashed lines.

Figure 1 shows a bottom-gate and top-contact structured P3HT-based transistor with an Al rotating gate. Flat PTFE substrates with thicknesses of 3, 5, and 8 mm were utilized as dielectric layers for the transistors and as friction surfaces for generating triboelectricity. Briefly, a 7 wt% solution of P3HT (Rieke Metals) dissolved in chloroform was deposited by spin coating on a plate at 2000 rpm for 30 s or by drop casting directly on the plate, after which the plates were thermally cured at 90 °C for 30 min. Subsequently, Ag paste was painted on both upper edges of the semiconductor layer, which were used as the source and the drain electrodes, in which a square active layer with a channel width (W) and length (L) of 1.7 cm was formed. The channel layer thickness of the spin-coated and drop-catsed devices was approximately 1.88 and 2.81 μm, respectively. An aluminum wheel with a smooth surface with a width of 1.7 cm was brought into contact with the surface of the insulating layer rather than the gate electrode.

The electrical characteristics of the fabricated transistors were verified using a Keithley 2400 SourceMeter. The drain voltage (*V_D_*) was applied through this SourceMeter between the source and drain electrodes. To replace the application of the gate voltage (*V_G_*), a wheel-shaped rotating gate was fixed to a motor and contacted with the dielectric layer of the fabricated devices. When the motor was rotated at randomly selected speeds of 500, 890, 1460, 1860, and 2300 rpm, the gate continuously rubbed the dielectric layer to generate triboelectric charges to replace *V_G_*. The drain current (*I_D_*) of the device was measured as a function of the *V_D_* applied to the frictionally generated channel. All *I_D_*s were measured in negative values owing to the negatively applied *V_D_*, but as the *I_D_* was expressed by extracting the current amplified by the triboelectric, it was expressed as positive values in the figures. The *I_D_*s of each device were expressed as an average value based on the measurements of several devices.

## 3. Results and Discussion

**Figure 2:**
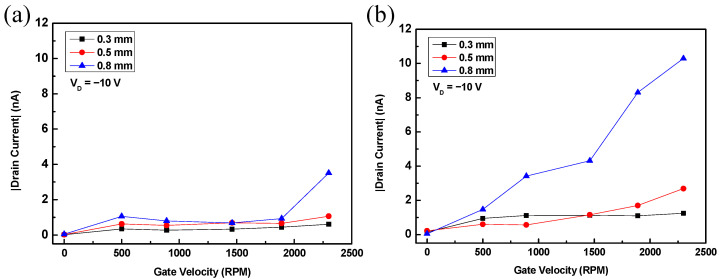
Transfer characteristics of the rotating gate transistor (RGTs) with active layers deposited using (**a**) spin-coating and (**b**) drop-casting techniques and dielectric layers of various thicknesses. The thickness of the dielectric layer of the device represented with squares, circles, and triangles in the graphs was 0.3, 0.5, and 0.8 mm, respectively.

The transfer characteristics of the fabricated RGTs are shown in Figure 2. In the experiment, *V_D_* was fixed at –10 V, and the velocity of the motor rotating the wheel-shaped Al gate was increased stepwise from 0 to 2300 rpm. The *I_D_* of the spin-coated RGT increased noticeably only at 2300 rpm at a dielectric layer thickness of 0.8 mm (Figure 2a). The *I_D_* curves of the drop-casted devices were relatively higher than those of the spin-coated devices (see Figure 2b). At a gate rotation velocity of 2300 rpm and a dielectric layer of 0.8 mm, the *I_D_* of the drop-casted device (10.297 nA) was approximately 2.93 times larger than that of the spin-coated device (3.515 nA). These transfer characteristic curves were very similar to those of conventional FETs; however, the *I_D_* of the proposed transistor improved with an increase in the thickness of the dielectric layer.

**Figure 3:**
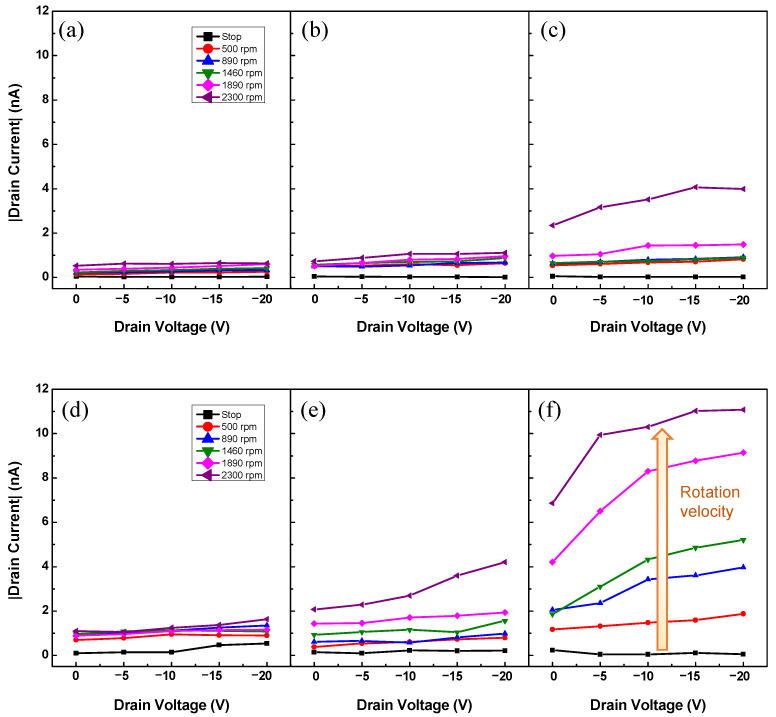
Output characteristics of the fabricated RGTs with active layers deposited by (**a**–**c**) spin-coating and (**d**–**f**) drop-casting. The dielectric layer thickness of each device in (**a**,**d**); (**b**,**e**); and (**c**,**f**) was 0.3, 0.5, and 0.8 mm, respectively.

Similarly, this phenomenon was also observed in the output characteristics (Figure 3). To measure the output characteristics, a wheel-shaped gate, in which the rotation velocity was increased stepwise from 0 to 2300 rpm, was utilized as in the transfer characteristics measurement, and *V_D_* was swept from 0 to –20 V at a step of 5 V. The results of the spin-coated devices are shown in Figure 3a–c, and those of the drop-casted devices are shown in Figure 3d–f. The *I_D_*s of both device types increased as the thickness of the dielectric layer increased. As the thickness of PTFE increases to a threshold value, more space is provided to release the deformation by external forces, which leads to an increase in current [32]. In addition, the output and transfer characteristics of the drop-casted and spin-coated devices were similar to those of conventional FETs, and the current amplification rates of the drop-casted devices were larger than those of the spin-coated devices. This is consistent with reports of conventional FETs ehxhibited higher *I_D_*s with an increase in the thickness of the semiconductor layers [33,34]. As the thickness of the semiconductor layer increased, the charge mobility (*μ*) of the transistor increased to a threshold value. The *I_D_* of the transistor changes according to *μ*, as expressed in the formula below [35,36]:

(1) $$I_{Dsat}=\frac{W}{2L}\mu C_{i}{\left(V_{G}-V_{T}\right)}^{2}$$

where *I_Dsat_* corresponds to *I_D_* in the saturation region, *C_i_* is the capacitance per unit area of the dielectric layer, and *V_T_* is the threshold voltage.

As illustrated in the graph, devices with drop-casted rather than spin-coated semiconductors and devices with thicker dielectric layers achieved higher current output. In addition, a steep increase in *I_D_* was observed in the spin-coated and drop-casted devices at a gate rotation velocity of 2300 and 1460 rpm, respectively (Figure 2). These characteristics indicated that the *I_D_*s of the fabricated devices are the output currents flowing through the channel formed in the semiconductor layer by friction between the gate and the dielectric.

**Figure 4:**
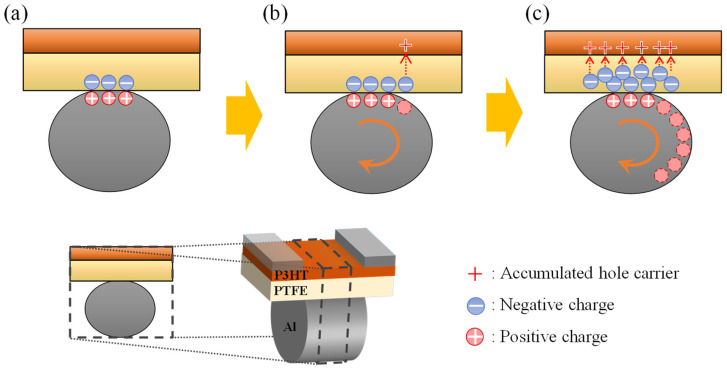
Theoretical schematic of channel generation by triboelectric charging in the fabricated RGT. (**a**) The Al gate and gateless device first contact, (**b**) the gate starts to rotate in the direction of the orange arrow and (**c**) rotates at high velocity. The circular plus (+) and minus (−) signs indicate the positive and negative charges generated via triboelectric charging by the rotating gate, respectively. The hole carriers induced by the negative charge (dotted arrow) of PTFE and accumulated at the semiconductor–insulator interface on the P3HT side are indicated by plus signs.

Figure 4 shows an illustration of the flow of electric charge assumed to have occurred on each layer during the rotation of the gate. First, when the wheel-shaped Al gate is in contact with the PTFE dielectric layer, creating negative and positive charges, respectively (Figure 4a). The gate is then rotated by the motor and leaves a negative charge on the PTFE only where it is separated from the PTFE. This negative charge induces a hole in the P3HT semiconductor layer (Figure 4b). As the rotation of the gate by the motor progressed, it continuously rubbed against the PTFE dielectric layer, leaving more negative charges and accumulating more hole carriers on the semiconductor surface (Figure 4c).

**Figure 5:**
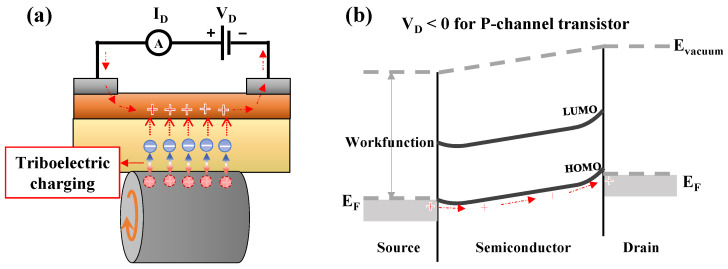
(**a**) Working mechanism of the fabricated RGT and (**b**) the energy level diagram along the channel of a p-channel transistor showing the transport of hole carriers from the source through the semiconductor to the drain. The orange arrow indicates the rotation of the gate, the circular plus (+) and minus (−) signs are positively and negatively charged triboelectric charges, respectively. The hole carriers indicated by plus signs induced from the negative charges flow along the dotted arrow and contribute to the *I_D_*. The work function of Ag used as the electrodes is 4.74 eV, which is adjacent to the highest occupied molecular orbital (HOMO) of P3HT, and the energy difference from the lowest unoccupied molecular orbital (LUMO) is large; thus, electrons are blocked [37,42,43].

Owing to the negatively-charged PTFE, hole carriers accumulated at the semiconductor–insulator interface inside the P3HT to form a channel, wherein the *I_D_* flowed using the hole carriers when a potential (*V_D_*) was applied between the drain and the source (Figure 5a). The fabricated RGT behaved partially differently from conventional FETs. In conventional FETs, the channel is formed by dielectric polarization via the application of voltage to the gate, whereas the channel was formed in the RGT by the triboelectric charging from the friction between the gate and the dielectric. Nevertheless, the characteristics of the FET in which the *I_D_* by the channel formed by the accumulation of charge carriers was also observed in the fabricated RGT. As shown in Figure 5b, when a negative voltage is applied to the drain electrode after holes are accumulated in the semiconductor to form a channel, the Fermi level (E_F_) of the drain electrode increases, thus facilitating the movement of holes from the source to the drain [37]. Furthermore, PTFE, which is a low-k material, does not readily form an electric field inside various dielectric materials and also tends to acquire a negative charge via contact between two different materials, whereas Al tends to gain a positive charge [38]. In conventional FETs, the generated output current decreases with an increase in the thickness of the dielectric layer and enters a saturation region at lower *V_D_*s, whereas in TENGs, the output voltage increases as the thickness of the dielectric layer increases before reaching the limit value [32,39,40,41]. In this study, the fabricated RGTs with a dielectric thickness of 0.8 mm exhibited the highest *I_D_*, followed by the devices with a dielectric thickness of 0.5 and 0.3 mm.

**Figure 6:**
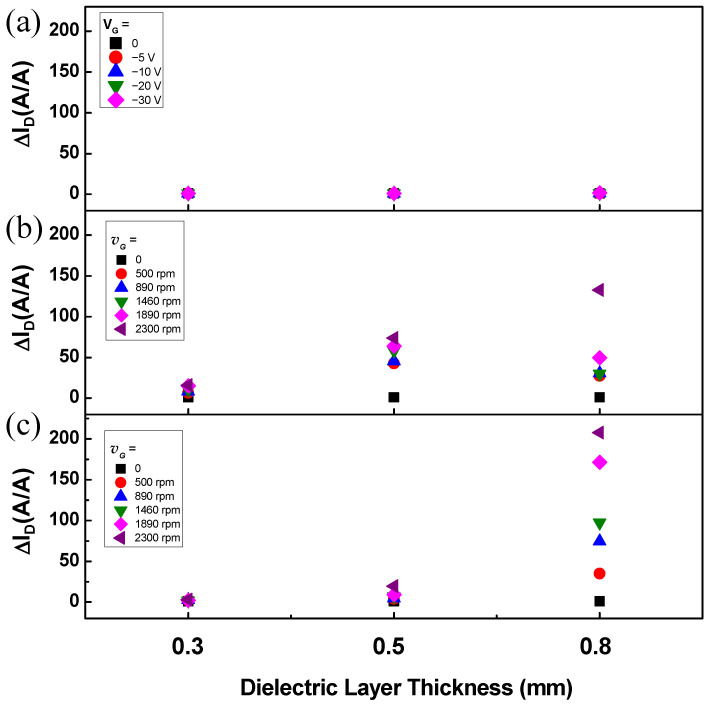
Comparison of the increase in the drain current (*I_D_*) according to (**a**) the gate voltage of a conventional transistor structure and (**b**,**c**) the gate rotation velocity (*v_G_*) of the fabricated RGT.

Figure 6 shows the *I_D_* on/off ratio (Δ*I_D_*) of transistors fabricated using the conventional FET structure and the fabricated RGTs. The transistor in Figure 6a was fabricated using the same method used to fabricate the RGT, but instead of the rotating Al gate in contact with the dielectric, a *V_G_* from 0 to –30 V was applied to the Al gate electrode deposited with a thickness of 40 nm under the PTFE layer. The FET structured transistor exhibited a remarkably low Δ*I_D_* even when a voltage of –30 V was applied to the gate (Figure 6a). As explained, this transistor was not expected to exhibit a field effect because PTFE was used as the dielectric layer, which restricts the formation of an internal electric field. In contrast, the *I_D_* of the fabricated RGT increased with an increase in the rotational velocity of the gate and the thickness of the dielectric layer (Figure 6b,c). However, devices with a dielectric layer thickness of 0.3 and 0.5 mm showed lower Δ*I_D_* in drop-casted devices with thicker channer layers than spin-coated devices. This is because a thicker channel layer with higher trap density results in an increase in the off-state *I_D_* (*I_Off_*) as expressed in the equation below [36]:

(2) $$I_{Off}=\frac{\sigma Wt}{L}V_{D}$$

where *σ* is the electrical conductivity, and *t* is the channel layer thickness. In contrast, the drop-casted device exhibited a higher Δ*I_D_* among devices with a dielectric layer of 0.8 mm owing to the significantly higher on-state *I_D_* despite the increase in *I_Off_*. Particularly, the spin-coated device with a dielectric layer thickness of 0.8 mm exhibited an Δ*I_D_* of 132.83 at a gate velocity of 2300 rpm, and the drop-casted device exhibited an Δ*I_D_* of 207.66.

## 4. Conclusions

In this study, we implemented a triboelectric-based RGT and investigated its electrical properties. The RGT exhibited the typical characteristics of typical triboelectric-based devices, in which the *I_D_* of the fabricated device increased with an increase in the gate velocity and thickness of the dielectric layer. Particularly, the *I_D_* of the fabricated RGT increased by 207.66 times at a gate rotation velocity of 2300 rpm compared to that at a stopped gate. In addition, the fabricated RGT with a similar structure to a conventional FET exhibited the triboelectric effect instead of the field effect. Similar to that of a conventional FET, the *I_D_* of the fabricated RGT was saturated at a certain *V_D_*, and the *I_D_* increased steeply at a certain gate rotation velocity. These results indicate that the fabricated RGT can not only exhibit the velocity of a motor as an electric current but can also be utilized to reduce energy consumption by harvesting triboelectricity. The results of this study will help to close the research gap between triboelectric transistors and FETs, and the implemented device can be directly applied to the motors and wheels of automobiles, as well as all rotating bodies used in industries, such as self-powered speedometers, switches, and amplifiers. 

However, PTFE, which was used as the dielectric layer of the fabricated RGT, exhibits extremely hydrophobic properties, poor adhesion, and uneven surface roughness [44,45]. In addition, the developed device was significantly larger than conventional FETs, and the thickness of the semiconductor film in the spin-coated device was approximately 1.88 μm, which may result in a poor switching behavior owing to high leakage current. In addition, P3HT may undergo oxidative doping depending on the experimental environment owing to its low ionization potential, which also results in an increase in leakage current [46,47]. The direction of our future research is to conduct an in-depth examination of voids or delaminations between the semiconductor and dielectric layers of RGTs and to address adhesion using methods, such as plasma surface treatment. Furthermore, through device downsizing and process optimization, leakage current can be minimized, and performance can be improved, so that the research results can be utilized in various applications, including self-powered speedometers and motor-driven switches [48,49].

## Data Availability

Not applicable.
